# Efficacy and safety of mepolizumab in a Chinese population with severe asthma: a phase III, randomised, double-blind, placebo-controlled trial

**DOI:** 10.1183/23120541.00750-2023

**Published:** 2024-05-20

**Authors:** Ruchong Chen, Liping Wei, Yuanrong Dai, Zaiyi Wang, Danrong Yang, Meiling Jin, Cui Xiong, Ting Li, Shuling Hu, Jie Song, Robert Chan, Subramanya Kumar, Azza Abdelkarim, Nanshan Zhong

**Affiliations:** 1 State Key Laboratory of Respiratory Disease, National Clinical Research Center for Respiratory Disease, National Center for Respiratory Medicine, Guangzhou Institute of Respiratory Health, the First Affiliated Hospital of Guangzhou Medical University, Guangzhou, China; 2 The 3rd Affiliated Hospital of Guangzhou Medical University, Guangzhou, China; 3 The 2nd Affiliated Hospital of Wenzhou Medical University, Wenzhou, China; 4 The First Affiliated Hospital of Xinjiang Medical University, Urumqi, China; 5 Shanghai 6th People's Hospital Affiliated to Shanghai Jiao Tong University School of Medicine, Shanghai, China; 6 Zhongshan Hospital Fudan University, Shanghai, China; 7 R&D, GSK, Shanghai, China; 8 Clinical Sciences Respiratory, R&D, GSK, Brentford, Middlesex, UK; 9 Safety Evaluation and Risk Management, GSK, Brentford, Middlesex, UK; 10 Safety Evaluation and Risk Management, GSK, Warsaw, Poland

## Abstract

**Background:**

In China, the prevalence of severe asthma with eosinophilic phenotype is rising, yet treatment options are limited. Mepolizumab is the first targeted biologic therapy for eosinophilic-driven disease in China. This study (clinicaltrials.gov identifier NCT03562195) evaluated efficacy and safety of mepolizumab in Chinese patients with severe asthma.

**Methods:**

The phase III, multicentre, randomised, placebo-controlled, double-blind, parallel-group study enrolled patients aged ≥12 years with severe asthma, with two or more exacerbations in the previous year, and on inhaled corticosteroids plus at least one controller medication. Following a 1–4-week run-in, patients were randomised 1:1 to mepolizumab 100 mg or placebo subcutaneously every 4 weeks for 52 weeks. The primary end-point was annualised rate of clinically significant exacerbations (CSEs) through week 52. Secondary end-points were time to first CSE, frequency of CSEs requiring hospitalisation/emergency department visits or hospitalisation over 52 weeks, mean change in St George's Respiratory Questionnaire (SGRQ) total score and pre-bronchodilator forced expiratory volume in 1 s (FEV_1_) at week 52; safety was evaluated.

**Results:**

The modified intention-to-treat population included 300 patients. At week 52 with mepolizumab *versus* placebo, annualised rate of CSEs was 65% lower (0.45 *versus* 1.31 events per year; rate ratio 0.35, 95% CI 0.24–0.50; p<0.001); time to first CSE longer (hazard ratio 0.38, 95% CI 0.26–0.56; p<0.001) and number of CSEs requiring hospitalisation/emergency department visit lower (rate ratio 0.30, 95% CI 0.12–0.77; p=0.012). From baseline to week 52, SGRQ score improved (p=0.001) and pre-bronchodilator FEV_1_ increased (p=0.006). Incidence of adverse events was similar between treatment groups.

**Conclusion:**

Mepolizumab provided clinical benefits to patients with severe asthma in China and showed a favourable benefit–risk profile.

## Shareable abstract

**Mepolizumab in a Chinese population with severe asthma reduced clinically significant exacerbations and improved lung function and quality of life with no new safety signals compared with placebo, supporting use for an unmet medical need in this population** https://bit.ly/47GZIDY

## Introduction

Severe asthma is defined by Chinese asthma guidelines and the Global Initiative for Asthma (GINA) as asthma requiring maximal optimised therapy of high-dose inhaled corticosteroids (ICS) and a second controller medication and/or oral corticosteroids (OCS) to achieve symptom control, or disease that remains uncontrolled despite these therapies [1–5]. Eosinophilic asthma is a phenotype characterised by induced eosinophilia in sputum (with an increase in peripheral blood eosinophils) or elevated fractional exhaled nitric oxide [6, 7]. Patients with severe asthma experience frequent exacerbations, poor disease control and health-related quality of life, and impaired lung function [3, 7]. In China, the prevalence of asthma in people aged ≥14 years is reported to be 1.24%, with ∼6% of asthmatic patients having severe disease, consistent with global rates (5–10%) [3, 8, 9]. Several biologics have been approved for severe asthma in multiple regions around the world [10]. In China, omalizumab is the only approved biologic targeting immunoglobulin E for moderate-to-severe allergic asthma; however, there are no biologics indicated for the eosinophilic phenotype [4]. Therefore, an effective and safe therapy is urgently needed to address the disease burden and optimise available treatments for patients with severe asthma in China.

Mepolizumab is a humanised monoclonal antibody that targets interleukin (IL)-5, the primary cytokine by which eosinophils respond to signals promoting differentiation, activation and survival [11–13]. Mepolizumab is approved for the treatment of severe asthma with an eosinophilic phenotype, among other indications in various regions worldwide; in China, mepolizumab is the first biologically targeted therapy to be approved for the treatment of eosinophilic-driven disease (for patients with eosinophilic granulomatous polyangiitis) [13–15]. Approval of mepolizumab for use in severe asthma with an eosinophilic phenotype in other regions was supported by several phase III studies, which demonstrated reduced exacerbations and OCS use, and improved asthma control, lung function (forced expiratory volume in 1 s (FEV_1_)) and health-related quality of life in patients who received mepolizumab compared to placebo [16–19]. In particular, the global study Mepolizumab adjUnctive therapy in subjects with Severe eosinophiliC Asthma (MUSCA; clinicaltrials.gov identifier NCT02281318) showed that mepolizumab decreased exacerbations by 58% [16]. Other studies including patients with severe asthma with an eosinophilic phenotype in Japan and South Korea have reported the clinical benefits of mepolizumab or benralizumab (which targets the IL-5 receptor) [20–23]. However, clinical investigation of mepolizumab in the Chinese population has not yet been assessed. Therefore, this phase III study, based on the study design of MEpolizumab as adjunctive therapy iN patients with Severe Asthma (MENSA; clinicaltrials.gov identifier NCT01691521), evaluated the efficacy and safety of mepolizumab 100 mg administered subcutaneously once every 4 weeks in Chinese patients with severe asthma. The efficacy and safety results of this study are reported here.

## Materials and methods

### Study design

The design of this 52-week study was modelled on the 32-week phase III MENSA study, featuring the same primary end-point, mepolizumab dose regimen, treatment schedule and disease phenotype. This was a multicentre, randomised, double-blind, placebo-controlled, parallel-group study of mepolizumab *versus* placebo conducted at 45 sites in China from August 2018 to September 2022 (clinicaltrials.gov identifier NCT03562195). The Consolidated Standards of Reporting Trials reporting guidelines were used in the development of this article [24].

This study consisted of a 1–4-week run-in period for collection of baseline data, followed by a 52-week treatment period (supplementary figure S1). Patients were stratified based on blood eosinophil count at screening (visit 1) (≥300 cells·µL^−1^ *versus* <300 cells·µL^−1^), with a minimum enrolment of 150 patients who had blood eosinophil counts ≥300 cells·µL^−1^. Patients who met eligibility criteria at screening then completed the run-in period. Those who experienced an asthma exacerbation remained in the run-in period until their baseline asthma status had been maintained for ≥1 week; any unable to achieve this did not enter the treatment period. Following the run-in period, at visit 2, patients were randomised 1:1 using an interactive web response system to receive placebo or mepolizumab 100 mg *s.c.* During the treatment period, sponsor and site staff were blinded to blood eosinophil count.

Double blinding of treatment groups was maintained throughout the treatment period (supplementary methods). Participants remained on their current standard-of-care maintenance therapy throughout the run-in and double-blind treatment periods. Given that unblinding of eosinophil counts may have compromised the integrity of the study, neither the site nor GSK personnel were sent results from the central laboratory for either absolute eosinophil counts or white blood count differentials.

From screening through their last visit, patients maintained a daily eDiary recording their morning peak expiratory flow in L·min^−1^ (best of three attempts) prior to use of any rescue medication, use of rescue medication in the previous 24-h period, frequency of awakening due to asthma symptoms requiring rescue medication and assessment of their asthma symptoms over the previous 24-h period based on a six-point scale. For the patients’ safety, automatic alerts were sent to investigators if pre-defined alert criteria indicative of worsening asthma were met and recorded in the eDiary.

The study was conducted in accordance with the ethical principles of the Declaration of Helsinki, the International Conference on Harmonization Good Clinical Practice guidelines, and any applicable country-specific regulatory requirements. All patients provided written informed consent. The study was approved by local ethics committees of the participating sites. The full listing of local approval committees and approval numbers are available upon request.

### Study subjects

Eligible patients were aged ≥12 years and weighed ≥40 kg. Patients had eosinophilic severe asthma with persistent airflow obstruction at visit 1 (age ≥18 years: pre-bronchodilator FEV_1_ <80% predicted [25]; age 12–17 years: pre-bronchodilator FEV_1_ <90% predicted or FEV_1_/forced vital capacity ratio <0.8). Severe asthma with an eosinophilic phenotype was identified by elevated peripheral blood eosinophil counts (related to asthma of either ≥300 cells·µL^−1^ within 12 months prior to study start or ≥150 cells·µL^−1^ at visit 1) in combination with criteria based on those from the American Thoracic Society (ATS) workshop on refractory asthma (2000) [26]. Prior to visit 1, patients must have received regular treatment with ICS (≥500 µg·day^−1^ fluticasone propionate or equivalent; an optimised therapeutic approach per clinical practice for severe asthma in China) for 12 months in total, and in the preceding 3 months, with or without maintenance OCS (≥5 mg prednisone or equivalent), plus at least one additional controller medication (besides ICS) used regularly for ≥3 months. Patients must also have experienced two or more exacerbations requiring treatment with systemic corticosteroids administered (*via* intravenous, intramuscular or oral routes) in the previous 12 months. Current smokers or former smokers (patients who quit smoking ≥6 months prior to visit 1) with a smoking history of ≥10 pack-years ((number of cigarettes per day/20)×number of years of smoking) were excluded. Additional inclusion, exclusion and randomisation criteria are provided in the supplementary methods.

### Methods

The primary end-point was the annualised rate of clinically significant exacerbations (CSEs) (events per year) of asthma over the 52-week treatment period. CSEs were defined as worsening of asthma which required the use of systemic corticosteroids and/or hospitalisations and/or emergency department (ED) visits; their occurrence was also verified using objective evidence from the eDiary (supplementary methods). Secondary end-points included time to first CSE and annualised rate of CSEs requiring hospitalisation (including intubation and admittance to intensive care unit) and/or ED visits and hospitalisation over the 52-week treatment period, and mean change from baseline in pre-bronchodilator FEV_1_ and St George's Respiratory Questionnaire total score at week 52.

Safety was assessed by monitoring of adverse events/serious adverse events (SAEs) (including adverse events of special interest (AESI), *i.e.* systemic/local reactions), haematological and clinical chemistry, vital signs and 12-lead ECG.

Blood eosinophil counts were recorded as part of standard haematological assessments. After visit 2, blood eosinophil counts were blinded to the sponsor and site staff.

### Analysis

All randomised patients who received at least one dose of treatment were included in the modified intention-to-treat (mITT) population. The per-protocol population, which included patients from the mITT population not identified as full protocol deviators (decided prior to unblinding), was used for sensitivity analysis of the primary end-point. Statistical analyses were performed using SAS version 9.4 or a later release.

A sample size of 300 patients was hypothesised to provide 90% power to detect a 40% decrease in the exacerbation rate using a two-sided 5% significance level, but after enrolling only 220 patients (due to coronavirus disease 2019 (COVID-19) pandemic conditions), a blinded evaluation indicated the exacerbation rate was lower than expected, reducing the power to 66%. Therefore, the study was amended from a fully powered bridging study to one using a Bayesian dynamic borrowing model [27] (BDB) to estimate the treatment effect for Chinese patients (mITT population) *via* the logarithmic rate ratio (mepolizumab:placebo) of CSEs by borrowing data from MENSA (ITT population). This Bayesian analysis used a robust mixture prior distribution [27], which allowed for “dynamic borrowing” of prior information. This analysis “learned” how much of the global prior information from MENSA to borrow based on the consistency between the data from the Chinese mITT population and prior global MENSA ITT population (irrespective of geographic region/race/ethnic origin), and updated the weight on the global prior data accordingly. The mixture prior distribution was constructed by two components: 1) an informative prior component based on the observed rate ratio from the global MENSA study (*i.e.* global prior); and 2) a vague prior component centred on a mean of zero (with variance scaled to represent information equivalent to one patient for each treatment arm), representing “no effect”; the resulting prior distribution had the form: weight×(component 1)+(1−weight)×(component 2) (supplementary methods). In the primary analysis, a prior weight of 50% is proposed for the informative component of the robust mixture prior, with the remainder of the weight (50%) placed on the vague component to reflect a conservative starting position regarding the assumed relevance of the global MENSA population to the Chinese mITT population, resulting in 88.6% power. A frequentist approach utilised the Chinese mITT population (formal hypothesis testing was not pre-planned in the protocol).

The primary end-point was analysed using a generalised linear model assuming a negative binomial distribution and adjusting for covariates used in MENSA [18]. A 95% posterior probability that the true rate ratio <1 represented a high level of confidence in declaring a positive treatment benefit with mepolizumab *versus* placebo. Subgroup analyses of the primary end-point were conducted to assess consistency of the effect of mepolizumab.

The primary analysis used a negative binomial model that assumed missing data from withdrawn patients were missing at random. A treatment policy approach was used for the supplemental estimand to examine the sensitivity of the results of the primary analysis to departures from the assumption that intercurrent event did not occur. This supplemental estimand was also tested for sensitivity analysis by comparing multiple imputation by missing at random or jump to reference models. A supportive “tipping point” analysis was conducted to assess the proportion of data borrowed from MENSA that was needed to declare evidence of a reduced rate of CSEs with mepolizumab compared with placebo in the mITT population.

All secondary end-points were assessed in the Chinese mITT population. Time to first CSE was analysed using a Cox proportional hazards model. Changes in SGRQ total score and pre-bronchodilator FEV_1_ at week 52 were analysed using mixed-model repeated measures, adjusting for covariates as described in MENSA [18]. Exacerbations requiring hospitalisation and/or ED visits or hospitalisation alone were analysed as per the primary end-point analysis.

Safety end-points were evaluated in the Chinese mITT population. Adverse events were categorised as “on-treatment” from the administration of the first dose through 28 days following the last dose received. Adverse events were summarised by system organ class and preferred term if they occurred at any time during the on-treatment window.

## Results

### Patient population

**FIGURE 1:**
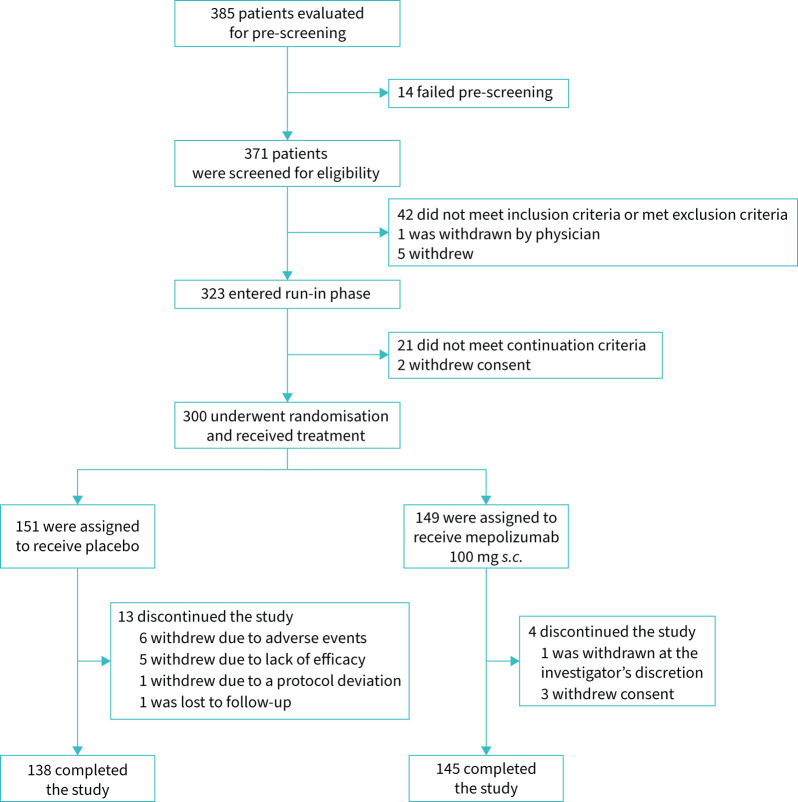
Patient disposition. *s.c.*: subcutaneous.

Of the 371 patients screened, 300 were randomised and included in the mITT population; of these, 283 (94.3%) completed the 52-week treatment period (figure 1). The most common reasons for study withdrawal were adverse events (n=6) and lack of efficacy (n=5), both in the placebo group. Notably, none of the withdrawals in either group were considered by the investigator to be COVID-19 related. The proportion of patients with treatment interruptions due to the COVID-19 pandemic was similar between mepolizumab (24.2%) and placebo groups (25.2%).

Baseline demographics, clinical characteristics and prior medication use were similar in the mepolizumab and placebo groups of the mITT population (table 1). Mean age was 52 years, with an average duration of asthma of 11.7 years. All patients had at least two exacerbations of their asthma in the 12 months prior to the study start, with a mean of 2.8 events. All patients initiated concomitant medication prior to study treatment including ICS, long-acting β-agonist, leukotriene receptor antagonist or corticosteroids. At baseline, 21 (7%) of patients received OCS with a mean±sd dose of 9.40±5.85 mg·day^−1^ (table 1). Overall, the mITT population was similar to the study population in MENSA, with a few exceptions (supplementary table S2).

**TABLE 1:** Baseline demographics and clinical characteristics (Chinese modified intention-to-treat population)

	**Placebo**	**Mepolizumab 100 mg *s.c.***	**Total**
**Patients**	151	149	300
**Age (years)**	53.7±13.59	50.8±12.59	52.2±13.16
**Female**	83 (55.0)	81 (54.4)	164 (54.7)
**Asian: East Asian heritage**	151 (100)	149 (100)	300 (100)
**BMI (kg·m^−2^)**	24.2±3.75	24.9±3.80	24.5±3.79
**Duration of asthma (years)**	12.2±11.46	11.3±10.64	11.7±11.05
**Baseline lung function**			
Pre-bronchodilation FEV_1_ (mL)	1543±701	1614±648	1578±675
Post-bronchodilation FEV_1_ (mL)	1708±689	1826±677	1767±684
Pre-bronchodilation FVC (mL)	2629±928	2776±854	2702±893
Pre-bronchodilation FEV_1_/FVC ratio	0.58±0.13	0.58±0.12	0.58±0.13
FEV_1_ reversibility (mL)	273±191	270±193	272±192
**Exacerbations in the prior 12 months**	2.8±1.29	2.8±1.48	2.8±1.39
0	0	0	0
1	0	0	0
2	88 (58.3)	91 (61.1)	179 (59.7)
3	34 (22.5)	29 (19.5)	63 (21.0)
4	15 (9.9)	15 (10.1)	30 (10.0)
>4	14 (9.3)	14 (9.4)	28 (9.3)
**OCS use at baseline**	12 (7.9)	9 (6.0)	21 (7.0)
Baseline OCS dose (mg·day^−1^)	10.6±6.92	7.70±3.74	9.40±5.85
**Current medical conditions** **^#^**			
Allergic rhinitis or hay fever	79 (52.3)	82 (55.0)	161 (53.7)
Hypertension	39 (25.8)	50 (33.6)	89 (29.7)
Sinusitis	20 (13.2)	27 (18.1)	47 (15.7)
Coronary artery disease	19 (12.6)	12 (8.1)	31 (10.3)
**Blood eosinophil count**			
≥300 cells·µL^−1^	87 (57.6)	87 (58.4)	174 (58.0)
<300 cells·µL^−1^	64 (42.4)	62 (41.6)	126 (42.0)
**Concomitant medication initiated before study treatment**			
ICS/LABA	151 (100)	149 (100)	300 (100)
SABA	84 (55.6)	81 (54.4)	165 (55.0)
LTRA	79 (52.3)	72 (48.3)	151 (50.3)
Corticosteroids^¶^	93 (61.6)	104 (69.8)	197 (65.7)
Other respiratory medication	36 (23.8)	43 (28.9)	79 (26.3)
**Smoking status**			
Current and former smokers (≥10 pack-years)	0	0	0
Former smokers (<10 pack-years)	31 (20.5)	23 (15.4)	54 (18.0)
Never-smokers	119 (78.8)	126 (84.6)	245 (81.7)

Data are presented as n, mean±sd or n (%). *s.c.*: subcutaneous; BMI: body mass index; FEV_1_: forced expiratory volume in 1 s; FVC: forced vital capacity; OCS: oral corticosteroid; ICS: inhaled corticosteroid; LABA: long-acting β-agonist; SABA: short-acting β-agonist; LTRA: leukotriene receptor antagonist. ^#^: current medical conditions present in ≥10% of all patients; ^¶^: including systemic, oral, parenteral and intra-articular.

### Primary end-point

As summarised in table 2, there was a significant reduction in the annualised rate of CSEs in the mepolizumab group *versus* placebo (>99.9% posterior probability that the rate ratio was <1) when analysed using the BDB approach. The annualised rate of CSEs was 65% lower with mepolizumab *versus* placebo (0.45 per year *versus* 1.31 per year; rate ratio 0.35, 95% CI 0.24–0.50; p<0.001) by the frequentist approach using data from the Chinese mITT population alone, which also supported the conclusion of the primary Bayesian analysis. The proportion of patients experiencing CSEs through week 52 was lower in the mepolizumab group (28.9%) compared with the placebo group (52.3%). A larger proportion of patients in the mepolizumab than the placebo group had ≥50% reduction in exacerbations from baseline (90.6% *versus* 70.2%) (supplementary table S3).

**TABLE 2:** On-treatment exacerbations through week 52

	**Placebo**	**Mepolizumab 100 mg *s.c.***
**Patients**	151	149
**Overview of exacerbations during the study (Chinese mITT population)**		
** **All exacerbations^#^		
Patients experiencing ≥1 event	81 (53.6)	43 (28.9)
Events	209	78
** **CSEs^¶^		
Patients experiencing ≥1 event	79 (52.3)	43 (28.9)
Events	203	73
Events treated with OCS	182	65
Days of OCS treatment per event treated with OCS	10.1±8.0	8.1±5.9
** CSEs requiring hospitalisation and/or ED visit**		
Patients experiencing ≥1 event	18 (11.9)	5 (3.4)
Events	23	7
Exacerbation rate (events per year)	0.10	0.03
Rate ratio, mepolizumab/placebo (95% CI)	0.30 (0.12–0.77)	
p-value	0.012	
** CSEs requiring hospitalisation**		
Number of patients experiencing ≥1 event	12 (7.9)	4 (2.7)
Number of events	15	5
Exacerbation rate (events per year)	0.05	0.02
Rate ratio, mepolizumab/placebo (95% CI)	0.32 (0.10–1.02)	
p-value	0.054	
**Analysis of rate of CSEs with mepolizumab *versus* placebo**		
** Exacerbation rate (events per year)**	1.31	0.45
** Frequentist approach** **^+^**		
Rate ratio, mepolizumab/placebo (95% CI)	0.35 (0.24–0.50)	
Log rate ratio±se, mepolizumab/placebo	−1.07±0.193	
p*-*value	<0.001	
** BDB approach** **^§^**		
Posterior mean (90% credible interval)	0.41 (0.31–0.51)	
Probability (rate ratio <1)^ƒ^	>0.999	

Data are presented as n, n (%) or mean±sd, unless otherwise stated. *s.c.*: subcutaneous; mITT: modified intention-to-treat; CSE: clinically significant exacerbation; OCS: oral corticosteroid; ED: emergency department; BDB: Bayesian dynamic borrowing. ^#^: CSEs and investigator-defined exacerbations (events not supported by any objective assessments were not included as CSEs, but included as investigator-defined exacerbations); ^¶^: defined as worsening of asthma which required the use of systemic corticosteroids and/or hospitalisations and/or ED visits, which was also a protocol-defined exacerbation; ^+^: using data from the Chinese mITT population alone; ^§^: pre-specified primary end-point using combined data from Chinese mITT population and the Mepolizumab as Adjunctive Therapy in Patients with Severe Asthma study; ^ƒ^: a positive outcome for mepolizumab was declared if the posterior probability of the rate ratio (mepolizumab/placebo) being <1 was >0.95.

Subgroup analyses suggested that the reduction in the rate of CSEs with mepolizumab compared with placebo was generally consistent regardless of baseline age, sex, weight, percentage predicted pre-bronchodilator FEV_1_, airway reversibility and OCS maintenance therapy, number of exacerbations in the year prior to screening and blood eosinophil counts (including the randomisation stratification factor) at screening (supplementary figure S2).

Sensitivity analyses using the supplemental estimand and comparing multiple imputation by missing at random or jump to reference models produced similar results to the primary analysis (supplementary figure S3A), indicating the robustness of the analytical method employed. To assess the robustness of the conclusion by the BDB approach, a tipping point analysis was performed; this analysis showed that for all credible intervals, the upper limit of the posterior 90% credible interval for the rate ratio was <1 (supplementary figure S3B), indicating a statistically significant treatment benefit of mepolizumab for the reduction of CSEs, regardless of the weight of data borrowing from MENSA, including the scenario under which no data were borrowed.

### Other end-points

**FIGURE 2:**
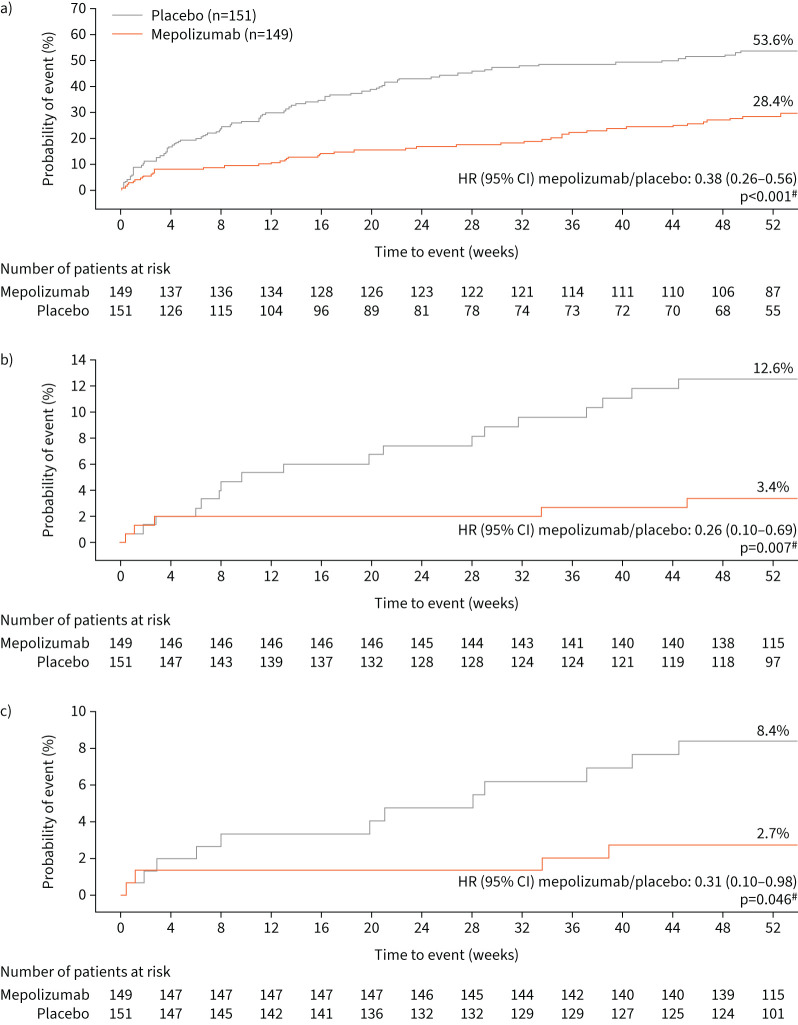
Kaplan–Meier plots of time to first a) clinically significant exacerbation (CSE), b) CSE requiring hospitalisation and/or emergency department (ED) visits and c) CSE requiring hospitalisation (Chinese modified intention-to-treat population only). HR: hazard ratio. ^#^: analysed using a Cox proportional hazards model, adjusting for covariates as described in the Mepolizumab as Adjunctive Therapy in Patients with Severe Asthma study [18].

Time to first CSE by week 52 was significantly longer in the mepolizumab group compared with the placebo group, as evidenced by the reduced probability of experiencing a CSE with mepolizumab by week 52 (28.4% in the mepolizumab group *versus* 53.6% in the placebo group) (hazard ratio (HR) 0.38, 95% CI 0.26–0.56; p<0.001; figure 2a). Similarly, time to first CSE requiring hospitalisation and/or ED visits was significantly increased with mepolizumab compared with placebo (HR 0.26, 95% CI 0.12–0.69; p=0.007; figure 2b), with lower probability of having a clinically significant exacerbation of this type by week 52: 3.4% for mepolizumab *versus* 12.6% for placebo (figure 2b). The annualised rate of CSEs requiring hospitalisation and/or ED visits was 0.03 per year with mepolizumab and 0.10 per year with placebo (rate ratio 0.30, 95% CI 0.12–0.77; p=0.012; table 2). A *post hoc* analysis of time to first CSE requiring hospitalisation alone followed a similar trend for mepolizumab benefit over placebo (HR 0.31, 95% CI 0.10–0.98; p=0.046), with lower probability of having a clinically significant exacerbation of this type by week 52: 2.7% for mepolizumab *versus* 8.4% for placebo (figure 2c). There was a 68% reduction in the annualised rate of CSEs requiring hospitalisation with mepolizumab *versus* placebo; however, this was statistically nonsignificant (0.02 per year *versus* 0.05 per year; rate ratio 0.32, 95% CI 0.10–1.02; p*=*0.054; table 2). Consistent with a higher incidence of CSEs in the placebo group compared with the mepolizumab group (203 events *versus* 73), patients who received placebo had a higher total number of days of OCS treatment for CSEs (1938 days) compared with those who received mepolizumab (489 days). The number of CSEs treated with OCS was also higher in the placebo group (n=182) compared with mepolizumab (n=65); the mean±sd number of days of OCS treatment per CSE treated with OCS was 8.1±5.9 days with mepolizumab and 10.1±8.0 days with placebo.

**FIGURE 3:**
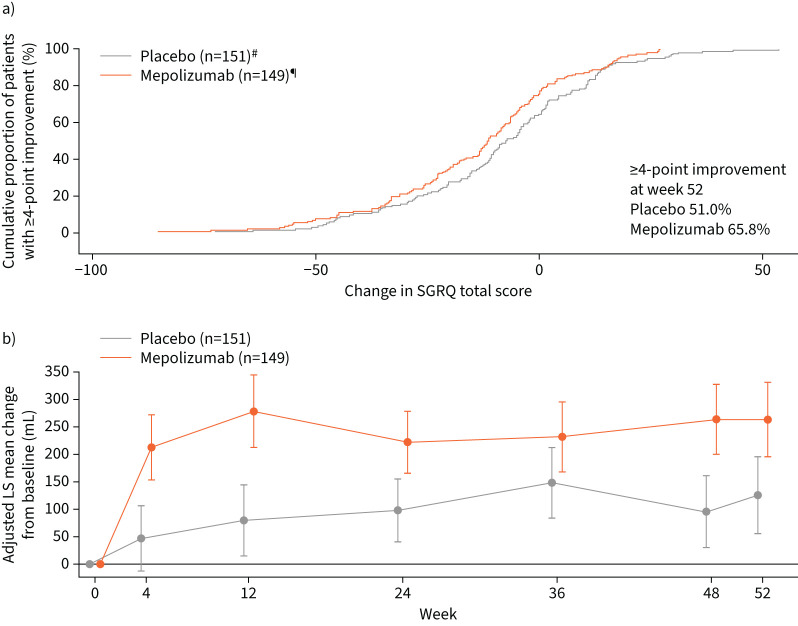
a) Cumulative distribution of patients with ≥4-point improvement from baseline in total St George's Respiratory Questionnaire (SGRQ) score at week 52. b) Pre-bronchodilator forced expiratory volume in 1 s (FEV_1_) change (95% CI) from baseline through week 52 (Chinese modified intention-to-treat population); placebo least-squares (LS) mean change±se at week 52: 125.67±35.49; mepolizumab LS mean change at week 52: 262.79±34.34; mean difference (95% CI) between mepolizumab and placebo at week 52: 137.13 (39.79–234.46), p=0.006 (analysed using mixed-model, repeated-measures method adjusting for covariates used in the Mepolizumab as Adjunctive Therapy in Patients with Severe Asthma study) [18]. ^#^: missing data: 18 (11.9%) out of 151; ^¶^: missing data: seven (4.7%) out of 149.

At baseline, mean total SGRQ scores were similar across the treatment groups (mepolizumab 38.9; placebo 39.1). At week 52, a greater mean decline (improvement) from baseline in SGRQ scores was observed with mepolizumab (−14.04) compared with placebo (−8.52); the intergroup treatment difference in SGRQ total score was −7.10 points in favour of mepolizumab (p*=*0.001). Furthermore, the cumulative proportion of patients with a ≥4-point improvement in SGRQ at week 52 was greater in the mepolizumab group than the placebo group (65.8% *versus* 51.0%; figure 3a).

Patients in both treatment groups had similar baseline pre-bronchodilator FEV_1_ values (table 1). Following treatment, significant improvements were seen with mepolizumab *versus* placebo as early as week 4 and at subsequent time points through week 52 (p=0.006) apart from week 36 (p*=*0.067; figure 3b). The least-squares mean increase from baseline to week 52 in pre-bronchodilator FEV_1_ was 263 mL with mepolizumab compared to 126 mL for placebo.

### Safety

The overall incidence of any on-treatment adverse events (90.6% *versus* 96.7%) and treatment-related adverse events (36.2% *versus* 41.1%) were slightly lower in the mepolizumab group compared with the placebo group (table 3). Adverse events that led to treatment discontinuation or study withdrawal occurred in one (<1%) patient in the mepolizumab group *versus* six (4.0%) in the placebo group.

**TABLE 3:** Summary of on-treatment adverse events^#^ (safety end-points) (Chinese modified intention-to-treat population)

	**Placebo**	**Mepolizumab 100 mg *s.c.***
**Patients n**	151	149
**Adverse events**		
Any on-treatment event	146 (96.7)	135 (90.6)
Treatment-related event	62 (41.1)	54 (36.2)
Leading to treatment discontinuation/study withdrawal	6 (4.0)	1 (<1)
**SAEs**		
Any on-treatment event	25 (16.6)	18 (12.1)
Treatment-related event	14 (9.3)	5 (3.4)
Resulting in death	2 (1.3)^+^	0
**Most common (≥5%) on-treatment adverse events** ^¶^		
Asthma	87 (57.6)	47 (31.5)
Upper respiratory tract infection	63 (41.7)	61 (40.9)
Nasopharyngitis	27 (17.9)	25 (16.8)
Bronchitis	16 (10.6)	17 (11.4)
Rhinitis allergic	15 (9.9)	12 (8.1)
Productive cough	11 (7.3)	11 (7.4)
Pneumonia	13 (8.6)	7 (4.7)
Headache	10 (6.6)	8 (5.4)
Cough	7 (4.6)	10 (6.7)
Dizziness	6 (4.0)	11 (7.4)
Pharyngitis	7 (4.6)	10 (6.7)
Rhinitis	5 (3.3)	10 (6.7)
Arthralgia	9 (6.0)	5 (3.4)
Oropharyngeal pain	4 (2.6)	9 (6.0)
**Most common (≥2%) on-treatment SAEs** ^¶^		
Asthma	12 (7.9)	4 (2.7)
Pneumonia	5 (3.3)	2 (1.3)
Nasal polyps	1 (<1)	3 (2.0)
**Systemic or local injection-site reactions**		
Systemic reactions	2 (1.3)	4 (2.7)
Local injection-site reaction	2 (1.3)	3 (2.0)
Anaphylaxis	0	0

Data are presented as n or n (%). *s.c.*: subcutaneous; SAE: serious adverse event. ^#^: any adverse events experienced during active study treatment; ^¶^: in either treatment group; **^+^**: due to central nervous system infection (n=1) and cause not identified (n=1).

The most frequently reported adverse events were asthma (mepolizumab 31.5%; placebo 57.6%), upper respiratory tract infection (mepolizumab 40.9%; placebo 41.7%) and nasopharyngitis (mepolizumab 16.8%; placebo 17.9%), with all other events reported in <15% of patients in either group (table 3).

SAEs were reported in 12.1% of the mepolizumab group and 16.6% of the placebo group; 3.4% and 9.3%, respectively, were considered by the investigator to be study-treatment related. The SAEs reported in three or more patients in either group were asthma (mepolizumab 2.7%; placebo 7.9%), pneumonia (mepolizumab 1.3%; placebo 3.3%) and nasal polyps (mepolizumab 2%; placebo <1%) (table 3).

There were two deaths during the study, which occurred in the placebo group and were considered study-treatment related; one patient died due to a central nervous system infection, while the other patient's cause of sudden death was not identified (table 3).

Consistent with the historic mepolizumab clinical development programme for patients with severe asthma, AESI included systemic and local injection-site reactions, serious cardiac, vascular and thromboembolic events, potential opportunistic infections and malignancies. The incidence of AESI was generally low and similar between the two treatment groups. “All infections” was the most frequently reported AESI, with similar incidences in both groups (mepolizumab 71.8%; placebo 74.2%). The incidence of systemic reactions was low and similar between both treatment arms (mepolizumab 2.7%; placebo 1.3%). Anaphylaxis was not reported in either group (supplementary table S1).

Mean laboratory clinical chemistry values and vital signs were generally similar across treatment groups at pre-treatment and post-baseline visits.

### Pharmacodynamics

Geometric mean blood eosinophil levels reduced from 405 cells·μL^−1^ at baseline to 50 cells·μL^−1^ by week 4 in the mepolizumab group and levels remained consistent to week 52 (47 cells·μL^−1^). The 84% reduction from baseline to week 52 was statistically significantly greater in the mepolizumab group compared with the placebo group (ratio 0.16, 95% CI 0.12–0.20; p<0.001). In the placebo group, blood eosinophil levels remained at baseline level (326 cells·μL^−1^) from week 4 (319 cells·μL^−1^) to week 52 (268 cells·μL^−1^).

## Discussion

Chinese guidelines and GINA are closely aligned on their definitions of eosinophilic phenotype [2, 5], while common unmet treatment needs have been identified across Chinese and European populations with severe asthma, including frequent exacerbations, poor symptom control and impaired quality of life despite treatment with high-dose ICS and/or OCS, as well as high rates of eosinophilic inflammation [28, 29]. This is the first phase III study to assess the safety and efficacy of mepolizumab among Chinese patients with severe asthma. Consistent with effects seen in the global population, mepolizumab was associated with reduced rate of CSEs and exacerbations requiring hospitalisations and/or ED visits, a longer time to CSE, as well as clinically meaningful improvements in health-related quality of life and improved lung function compared with placebo. The safety profile for mepolizumab was similar to that seen in previous studies, and no new safety signals were observed.

In this study, the primary end-point was met, demonstrated by a significant reduction in the rate of CSEs for patients receiving mepolizumab *versus* placebo. The use of the BDB approach for the primary end-point was permitted by the similarity in the study design between this and the MENSA study, as well as the predicted similarity in treatment effects. Unlike standard Bayesian approaches which borrow a fixed amount of prior information, dynamic borrowing is able to account for inconsistencies by learning how much information to borrow and making statistical adjustments, *i.e.* most data are borrowed when the current data are consistent with external data and less are borrowed when the current data are inconsistent. The tipping point analysis indicated that borrowing of MENSA data was not needed to achieve statistical significance. Similarly, frequentist analysis using data from this study alone also found a significant reduction in the frequency of CSEs with mepolizumab. Interestingly, the reduction in the rate of CSEs with mepolizumab 100 mg *s.c.* in this study exceeded that reported from MENSA (65% *versus* 53%) [18], although direct comparative analysis of these data was not performed. Differences in baseline characteristics of the patient population may have contributed; for example, patients in MENSA had a longer asthma history of ∼20 years *versus* 11.7 years, a higher number of exacerbations in the past year (3.5–3.8 severe events *versus* 2.8 exacerbations) and a higher proportion of patients receiving OCS maintenance treatment (∼27% *versus* 7.7% of patients) than those in the current study. Overall, this suggests that patients in the current study had less severe disease than in MENSA. Conversely, a greater proportion of patients in the Chinese population who received mepolizumab had a baseline blood eosinophil count ≥300 cells·µL^−1^ compared with MENSA (58% *versus* 53%) [30], suggesting potentially greater eosinophilic inflammation in the Chinese population, which may have contributed to the differences in CSE reduction between the two studies. This is consistent with findings from subgroup analysis of the MENSA study that demonstrated patients with the highest blood eosinophil counts (*i.e.* ≥500 cells·µL^−1^) had the greatest reduction in CSEs with mepolizumab [18]. However, it should be noted that the higher baseline blood eosinophil count thresholds were not assessed in the current study, unlike in the MENSA *post hoc* analysis [30]. Together, these findings provide an initial indication that earlier mepolizumab intervention in Chinese patients with greater eosinophilic inflammation may be clinically beneficial.

Rates of CSEs were lower with mepolizumab treatment *versus* placebo across all tested subgroups, including age, sex, weight, baseline predicted pre-bronchodilator FEV_1_, number of exacerbations in the year prior to screening and airway reversibility. Rate ratios for mepolizumab/placebo were highest in patients with baseline weight >75 kg (0.65) and pre-bronchodilator FEV_1_ >80% predicted (0.53), but remained well below 1. Rates of CSEs were also lower in the mepolizumab group irrespective of baseline blood eosinophil count, suggesting that mepolizumab was effective in Chinese patients with severe asthma of an eosinophilic phenotype regardless of their eosinophil levels. There was no real trend for greater efficacy with higher blood eosinophil counts, unlike in MENSA. Overall, the CSE results indicate the robustness of mepolizumab's clinical benefit across different patient populations and background demographic and clinical characteristics.

In regard to the secondary end-points, the clinical benefit observed with mepolizumab *versus* placebo was maintained, as demonstrated by improved time to first CSE, rate of exacerbations requiring hospitalisation, SGRQ score and change from baseline in pre-bronchodilator FEV_1_. The rate of CSEs requiring hospitalisation (excluding ED visits) was also reduced in the mepolizumab group; however, this was the only secondary end-point reported that did not reach statistical significance, which may be attributed to the low number of events across both groups. Overall, these results largely reflect the treatment difference between mepolizumab and placebo reported from MENSA and other phase III studies [16–18]. Therefore, this study adds to the body of literature supporting the efficacy of mepolizumab in patients with severe asthma.

The safety findings of mepolizumab observed in Chinese patients in this study is generally consistent with the known safety profile of mepolizumab [16–19]. The incidence of any on-treatment adverse events and treatment-related adverse events were similar between the mepolizumab and placebo groups. The incidence of SAEs was marginally higher in the placebo group compared with mepolizumab while AESI were generally low frequency, with systemic reactions and local site reactions affecting <5% of patients and no reports of anaphylaxis (supplementary table S1). Overall, the benefit–risk profile of mepolizumab appears to be favourable for patients with severe asthma in China.

The primary limitations of this study included the inability to directly compare results between Chinese and Western patients in the same trial. The study required that eligible patients had persistent airflow obstruction and recent exacerbations requiring treatment and excluded patients with concurrent respiratory disease. Although these study requirements were designed to ensure accurate interpretation of the study results, they do not necessarily reflect real-world clinical practice; however, such restrictions on eligibility criteria would be expected in phase III trials of patients with severe asthma. Evidence from global and European observational/registry studies demonstrates that clinical benefit with mepolizumab in patients with severe asthma in real-world settings is consistent with randomised controlled trials [31–33]. In addition, like many clinical trial programmes conducted during the COVID-19 pandemic, this study experienced challenges in recruiting patients and conducting site visits. The BDB approach was implemented to allow for analysis of the primary end-point and sensitivity analyses with sufficient power under the impact of COVID-19. A sensitivity analysis that evaluated the robustness of the BDB approach on outcomes showed that the degree of borrowing from MENSA did not affect treatment benefit with mepolizumab (assessed by rate ratio), including the scenario of note under which no data was borrowed from MENSA, and thus, did not contribute to bias. Also, a lower-than-expected number of exacerbation events was observed, which may be attributed to nonpharmacological interventions imposed by the pandemic, such as wearing masks, limiting social activities, lower probability of contracting seasonal flu or common cold, limiting physical activity and limiting time spent out of the home.

### Conclusion

Mepolizumab reduced CSEs and increased health-related quality of life and lung function compared with placebo in Chinese patients with severe asthma. No emerging safety issues specific to the Chinese patients were observed in this study and overall safety results were consistent with findings from previous clinical trials and the known safety profile of mepolizumab [16–19]. The findings of this study support the benefit of mepolizumab treatment and highlight the potential to address the severe unmet need of patients with severe asthma in China.

## Supplementary material

10.1183/23120541.00750-2023.Supp1 **Please note:** supplementary material is not edited by the Editorial Office, and is uploaded as it has been supplied by the author.
Supplementary material 00750-2023.SUPPLEMENT
