# Supplementary material for: Efficacy and safety of mepolizumab in a Chinese population with severe asthma: a phase III, randomised, double-blind, placebo-controlled trial
Source: ERJ Open Res. 2024 May 20;10(3):00750-2023. doi: 10.1183/23120541.00750-2023 (PMC11103715; doi:10.1183/23120541.00750-2023)
Supplement: Supplementary file 1 [file 00750-2023.SUPPLEMENT.pdf]

Efficacy and safety of mepolizumab in a Chinese population with severe asthma: A Phase III, randomized, double-blind, placebo-controlled trial

Ruchong Chen, MD;[1] Liping Wei, MD;[2] Yuanrong Dai, MD;[3] Zaiyi Wang, BSc;[4] Danrong Yang, MD;[5] Meiling Jin, MD;[6] Cui Xiong, PhD;[7] Ting Li, PhD;[7,*] Shuling Hu, MD;[7] Jie Song, MD;[7] Robert Chan, MD;[8] Subramanya Kumar, MRCP;[9] Azza Abdelkarim, PharmD;[10] Nanshan Zhong, MD[1]; on behalf of the 201536 study group

Supplementary Materials

Supplementary Methods

Eligibility criteria

Additional inclusion criteria:

- Documented peripheral blood eosinophil count of ⩾300 cells/μL in the prior 12 months OR 150 cells/μL at screening.
- Exacerbation history: for patients already receiving maintenance OCS, treatment for exacerbation had to require a ⩾2-fold increase in daily dosage for ⩾3 days

Additional exclusion criteria:

- Current or former smoking history (⩾10 pack years) or concurrent clinically significant respiratory disease that the investigator advised may affect the patient's asthma.
- Patients with current or personal history of cancer in remission for <12 months prior to screening (excluding resected localized skin carcinoma) or any history of lung cancer regardless of remission status.
- Abnormal chest X-ray or computed tomography scan not due to asthma, or bronchial thermoplastic and radiotherapy in the 12 months prior to Visit 1.
- Abnormal or unstable organ status, such as liver or biliary disease (alanine aminotransferase >2x upper limit of normal [ULN] or bilirubin >1.5x ULN), clinically significant, uncontrolled cardiovascular disease, abnormal electrocardiogram with corrected QT interval (QTc) prolongation, or any other concurrent, uncontrolled medical conditions that were deemed clinically significant in the opinion of the investigator.
- Other conditions associated with elevated eosinophils or immunodeficiency
- Immediate family members of the investigator, sub-investigator, or study employees, or had any personal history, such as substance abuse, psychiatric disease, biologic hypersensitivity, pregnancy or intellectual deficiency, that may interfere with study conduct.
- Patients were permitted to continue their baseline therapy, with the exception of the following: omalizumab within 130 days, other monoclonal antibodies to treat inflammatory disease within 5 half-lives, investigational drugs within 30 days or 5 half-lives (whichever is longer), herbal substances with the possibility of interfering with the safety of the patient's enrolment within 7 days, immunosuppressive drugs used to treat conditions other than asthma with variable washout periods, or prior participation in a clinical trial with mepolizumab.

Randomization criteria

Patients needed to fulfil the following criteria before being randomized into the study:

- Absence of clinically significant findings in the laboratory screening tests.
- Screening hepatitis B virus DNA level <2000 IU/mL.
- Evidence of asthma, as documented by one of the following:
    - Airway reversibility (forced expiratory volume in one second [FEV_1] $\geq$12% and 200 mL) at Visit 1 or Visit 2 or in the 12 months prior to Visit 2
    - Airway hyper-responsiveness (PC_{20} of <8 mg/mL or PD20 of <7.8 μmol methacholine/histamine) in the 12 months prior to Visit 2
    - Airflow variability in clinic FEV_1 $\geq$20% between two clinic visits in the 12 months prior to Visit 2 or >20% diurnal variability in peak flow observed on $\geq$3 days during the run-in
- eDiary compliance defined as completion of symptom scores, rescue medication information, and peak expiratory flow measures on $\geq$4 days out of the previous 7 days preceding Visit 2.
- No ongoing asthma exacerbation or changes in baseline IV corticosteroid or additional controller medication during run-in.

Double- blinding process

For the double- blinding process, mepolizumab and placebo were prepared by a designated, independent, unblinded member of the study site staff to be identical in appearance, then administered by a blinded member of the site staff. Treatment codes could be unblinded only in the case of a medical emergency or SAE, in which knowledge of the product administered was essential for the welfare of the patient, or to adhere to regulatory responsibilities for timely reporting of adverse AEs.

Bayesian Dynamic Borrowing model

The informative (global) prior component of the BDB was obtained from the sampling distribution of the log rate ratio between mepolizumab 100 mg SC and placebo in MENSA. The mean log rate ratio and its associated standard error were -0.7474 and 0.1532, respectively, providing a normal distribution with a mean of -0.7474 and standard deviation (SD) of 0.1532 as the global prior component for the primary treatment comparison. The vague prior component representing two patients (one in each treatment arm) assumed no treatment effect in the Chinese mITT population would be specified, to allow for the possibility that MENSA data did not provide relevant information about the treatment effect in Chinese patients. A normal distribution with a mean of zero and SD of 2.1256 was used as a weak prior distribution for log rate ratio.

Endpoints and assessments

Use of systemic corticosteroids in these cases was defined as requiring IV or OCS for $\geq$3 days or a single dose of IM corticosteroid, or at least double the existing dose for at least 3 days in patients on maintenance OCS (average daily dose of $\geq$5 mg prednisone or equivalent). CSEs recorded in the electronic case report form were verified using objective evidence from the eDiary to confirm that the exacerbation was associated with changes in PEF, rescue medication use, nocturnal awakening due to asthma, symptoms requiring rescue medication use or symptoms.

Supplementary Figures

Supplementary Figure 1. Study design

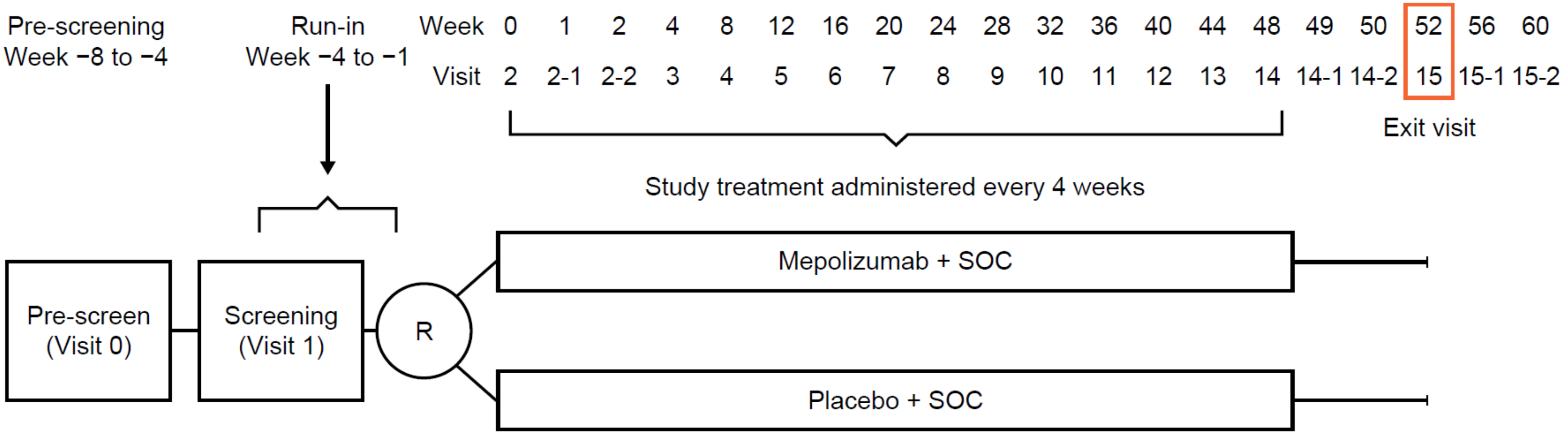

Q4W, every 4 weeks; R, randomization; SOC, standard of care

Supplementary Figure 2. Subgroup analyses of the rate of CSEs (Chinese mITT population)

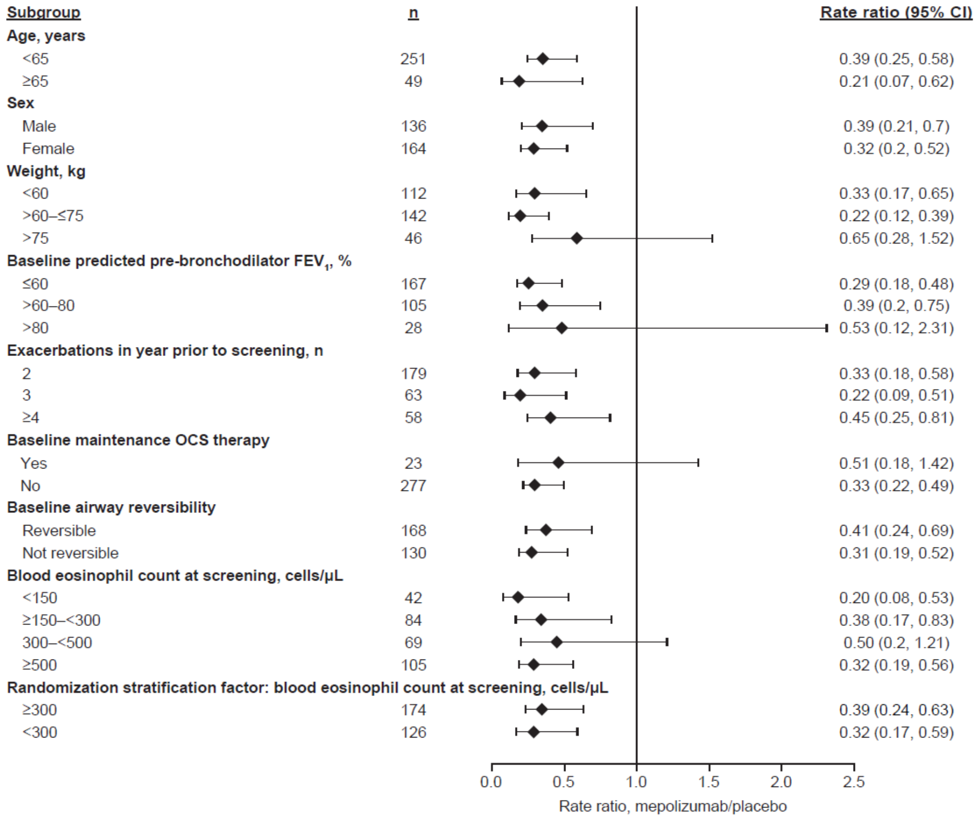

CI, confidence interval; CSE, clinically significant exacerbation; FEV_1, forced expiratory volume in one second; mITT, modified intent-to-treat; OCS, oral corticosteroid.

Supplementary Figure 3. (a) Sensitivity analyses of the primary endpoint (rate of CSEs) of the per-protocol population to assess the impact of different missing data models and (b) tipping point analysis to indicate the proportion of data borrowed from MENSA that was needed to declare evidence of reduced rate of CSEs in mITT population

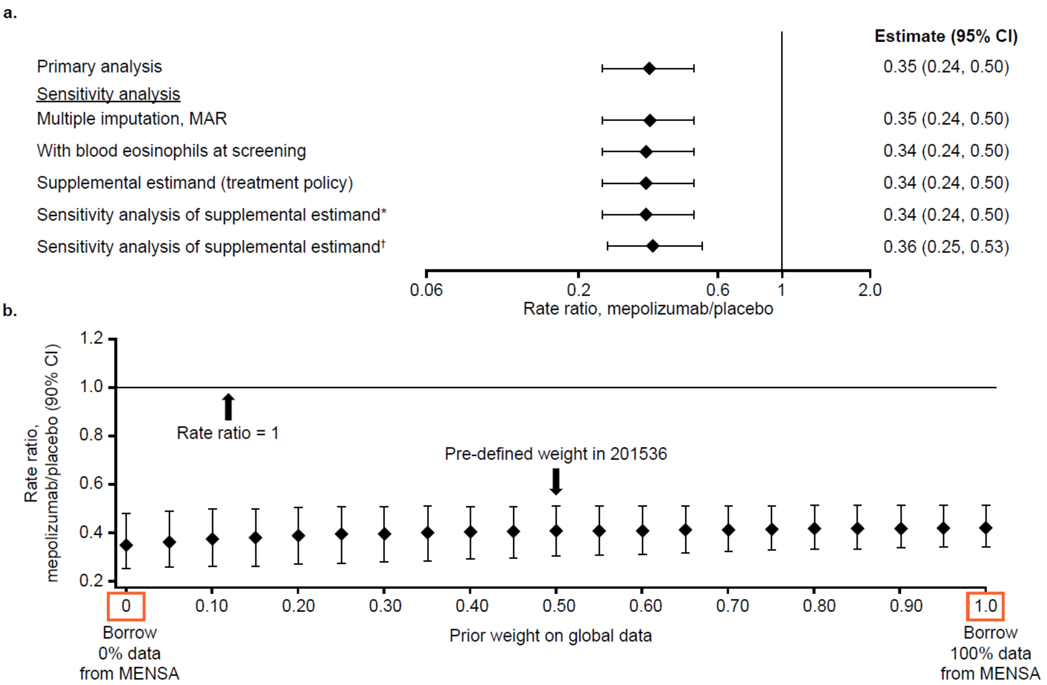

*Treatment policy, multiple imputation, MAR; †Treatment policy, multiple imputation, J2R.
CI, confidence interval; CSE, clinically significant exacerbation; J2R, jump to reference; MAR, missing at random; MENSA, Mepolizumab as Adjunctive Therapy in Patients with Severe Asthma; mITT, modified intent-to-treat.

Supplementary Table

Supplementary Table 1. Summary of on-treatment SAE and AESI (Chinese mITT population)

**SAEs and AESI, n (%)**	**Placebo (N=151)**	**Mepolizumab 100 mg SC (N=149)**	**Relative Risk[*] (95% CI)**
SAEs	25 (16.6)	18 (12.1)	0.73 (0.42, 1.28)
Systemic Reactions	2 (1.3)	4 (2.7)	2.03 (0.38, 10.90)
Anaphylaxis	0	0	–
Local Site Reactions	2 (1.3)	3 (2.0)	1.52 (0.26, 8.97)
All Infections[†]	112 (74.2)	107 (71.8)	0.97 (0.84, 1.11)
Serious Infections	11 (7.3)	5 (3.4)	0.46 (0.16, 1.29)
Opportunistic Infections[‡]	3 (2.0)	2 (1.3)	0.68 (0.11, 3.99)
Neoplasms[†]	4 (2.6)	2 (1.3)	0.51 (0.09, 2.72)
Malignancies[§]	0	0	–
Cardiac Disorders[†]	12 (7.9)	7 (4.7)	0.59 (0.24, 1.46)
Serious Cardiac Disorders	2 (1.3)	1 (<1)	0.51 (0.05, 5.53)
Serious CVT Events[**]	2 (1.3)	2 (1.3)	1.01 (0.14, 7.10)
Serious Ischemic Events[††]	0	2 (1.3)	–

Safety endpoints were evaluated in the Chinese mITT population. AEs were categorized as 'on-treatment' from the administration of the first dose through 28 days following the last dose received. [*]Relative risk between mepolizumab and placebo; [†]Infections from Infections and infestations SOC. Neoplasms from Neoplasms benign malignant and unspecified (including cysts and polyps) SOC. Cardiac disorders from Cardiac disorders SOC; [‡]Identified from SMQ or events with the preferred term of Herpes Zoster; [§]Identified from SMQs; [**]Serious CVT events identified from Cardiac disorders SOC, Vascular disorders SOC and SMQs; [††]Subset of Serious CVT events identified through SMQs.

AESI, adverse events of special interest; CI, confidence interval; CVT, Cardiac Vascular & Thromboembolic; SAE, serious adverse event; SC, subcutaneous; SOC, System Organ Class; SMQ, Standardized MedDRA query.

Supplementary Table 2. Study population characteristics in the Chinese mITT population compared with the MENSA trial[1].

	**Chinese patients with SA (NCT03562195)**	**MENSA (NCT01691521)**
Asian/East Asian, n (%)	100%	17%–20%
Former smoker, n (%)	Current or former smokers were excluded.	26%–30%
Prior CS use	Regular treatment with ICS (≥500 µg/day fluticasone propionate or equivalent, an optimized therapeutic approach per clinical practice for SA in China) for 12 months in total, and in the 3 months preceding, with or without maintenance OCS (≥5 mg prednisone or equivalent), plus ≥1 additional controller medication (besides ICS) used regularly for at least 3 months.	Treated with systemic glucocorticoids for ≥2 exacerbations in addition to treatment with ICS (≥880 µg of fluticasone propionate or the equivalent per day) and least 3 months of treatment with an additional controller.
OCS use at baseline, n (%)	7%	23%–27%
Baseline OCS dose, mg/day, mean	9.40	12.0–15.1

CS, corticosteroid; ICS, inhaled corticosteroid, OCS, oral corticosteroid, SA, severe asthma.

[1]Ortega HG, Liu MC, Pavord ID, Brusselle GG, FitzGerald JM, Chetta A, et al. Mepolizumab treatment in patients with severe eosinophilic asthma. N Engl J Med. 2014;371:1198-1207

Supplementary Table 3. Summary of Reduction in Exacerbations from Baseline (Chinese mITT Population)

	Placebo (N=151) n(%)	Mepolizumab 100 mg SC (N=149) n(%)
Increase	22 (14.6)	4 (2.7)
0-<10% Reduction	14 (9.3)	5 (3.4)
10-<20% Reduction	0	0
20-<30% Reduction	1 (<1)	1 (<1)
30-<40% Reduction	7 (4.6)	2 (1.3)
40-<50% Reduction	1 (<1)	2 (1.3)
>=50% Reduction	106 (70.2)	135 (90.6)
Cumulative Number of Subjects With:		
>=10% Reduction	115 (76.2)	140 (94.0)
>=20% Reduction	115 (76.2)	140 (94.0)
>=30% Reduction	114 (75.5)	139 (93.3)
>=40% Reduction	107 (70.9)	137 (91.9)
>=50% Reduction	106 (70.2)	135 (90.6)
